# Autophagy-Related Direct Membrane Import from ER/Cytoplasm into the Vacuole or Apoplast: A Hidden Gateway also for Secondary Metabolites and Phytohormones?

**DOI:** 10.3390/ijms15057462

**Published:** 2014-04-29

**Authors:** Ivan Kulich, Viktor Žárský

**Affiliations:** 1 Department of Experimental Plant Biology, Faculty of Sciences, Charles University, Viničná 5, 128 43 Prague 2, Czech Republic; E-Mail: kulich@natur.cuni; 2 Institute of Experimental Botany, Academy of Sciences of the Czech Republic, Rozvojová 263, 165 02 Prague 6, Czech Republic

**Keywords:** autophagy, ER stress, ER body, autophagosome, ATG proteins, exocyst, anthocyanins, vacuole, secondary metabolites, salicylic acid, phytohormones, exosomes

## Abstract

Transportation of low molecular weight cargoes into the plant vacuole represents an essential plant cell function. Several lines of evidence indicate that autophagy-related direct endoplasmic reticulum (ER) to vacuole (and also, apoplast) transport plays here a more general role than expected. This route is regulated by autophagy proteins, including recently discovered involvement of the exocyst subcomplex. Traffic from ER into the vacuole bypassing Golgi apparatus (GA) acts not only in stress-related cytoplasm recycling or detoxification, but also in developmentally-regulated biopolymer and secondary metabolite import into the vacuole (or apoplast), exemplified by storage proteins and anthocyanins. We propose that this pathway is relevant also for some phytohormones’ (e.g., auxin, abscisic acid (ABA) and salicylic acid (SA)) degradation. We hypothesize that SA is not only an autophagy inducer, but also a cargo for autophagy-related ER to vacuole membrane container delivery and catabolism. ER membrane localized enzymes will potentially enhance the area of biosynthetic reactive surfaces, and also, abundant ER localized membrane importers (e.g., ABC transporters) will internalize specific molecular species into the autophagosome biogenesis domain of ER. Such active ER domains may create tubular invaginations of tonoplast into the vacuoles as import intermediates. Packaging of cargos into the ER-derived autophagosome-like containers might be an important mechanism of vacuole and exosome biogenesis and cytoplasm protection against toxic metabolites. A new perspective on metabolic transformations intimately linked to membrane trafficking in plants is emerging.

## 1. Introduction: Intravacuolar Neutral Red Stained Bodies Have an Autophagic Origin

Major progress was achieved over the last two decades in the understanding of membrane and cargo transport to the vacuole; this certainly is the best studied domain of the endomembrane trafficking in plants [1–4]. Yet, even in this field, there are big outstanding questions, such as, e.g.,: what is the status and “ontogenesis” of two (in some cases, possibly more) different types of vacuoles in the same cell; how many membrane transport pathways connect to the tonoplast, and how are they regulated [5]?

Here, we will focus on recently emerging direct endoplasmic reticulum (ER) to vacuole pathway mediated by autophagy-like membrane transport containers, which transport cargo to the vacuole, bypassing the conventional Golgi-trans-Golgi network (GA-TGN) pathway. We use the category of “autophagy-like” or “autophagy-related” mechanisms to make a distinction from the concepts of the basal *vs*. induced autophagy or general *vs*. selective autophagy (mostly the organellar degradation pathway) as defined by [6] to highlight the possible basal housekeeping membrane and cargo trafficking functions of the autophagy-related pathway between ER and vacuole in plant cells.

**Figure 1.**
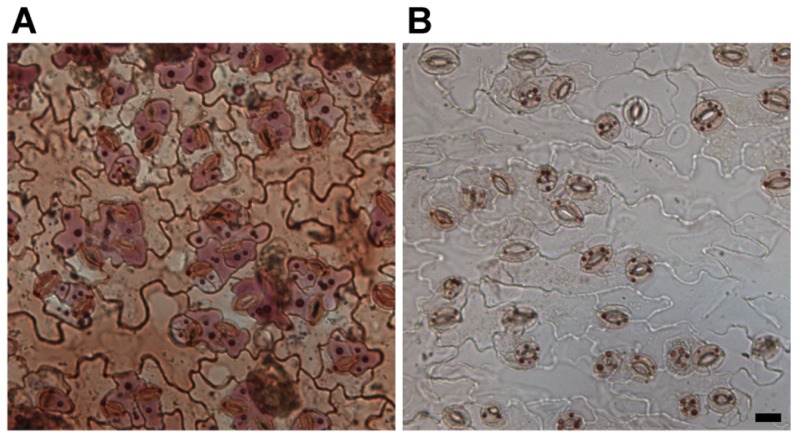
Neutral red staining of mature leaves that accumulate anthocyanins. Neutral red stained bodies (NRSBs) are larger and more abundant in anthocyanin accumulating cells of wild type (WT) (**A**); and (**B**) both NRSBs and anthocyanins are almost absent from the *Exo70B1-2* mutant. Scale bar is 20 μm long.

Plant vacuoles are known to contain many large intravacuolar bodies, including membranous ones. These can be stained by neutral red dye and have been named neutral red stained bodies (NRSBs). Despite many efforts, the origin and function of these compartments remains mysterious. As demonstrated by Figure 1A, NRSBs are quite large and can represent a significant volume transported into the vacuole. What is also apparent is that NRSBs are much larger in the anthocyanin accumulating cells (here, around the stomata). It has been shown before that these bodies are related to anthocyanin vesicular inclusions (AVIs) [7]. In Figure 1B, we can see an *Arabidopsis* vesicle tethering complex subunit *exo70B1-2* mutant, lacking both NRSBs and anthocyanins in the central vacuole.

The origin of the NRSBs was indicated to rely on autophagy processes, as the mutants in the autophagic pathway display decreased accumulation of both NRSBs and anthocyanins [7–9]. If we put these observations together with the reports on GA-independent import from ER into the vacuole (recently reported by [10,11]), it becomes apparent that, except for conventional pathways for vacuolar trafficking via GA, plants have developed a novel mechanism, which is adjusted to the enormous size of the vacuole and the extreme amounts of secondary metabolite cargoes. In many species, intravacuolar bodies contain large amounts of anthocyanins and are termed anthocyanin vesicular inclusions (AVIs). The literature on the trafficking of anthocyanins represents an important insight into the biology of intravacuolar bodies in general.

## 2. Anthocyanins and the Anthocyanin Vesicular Inclusions

Anthocyanins are secondary flavonoids, synthesized at the cytoplasmic site of the ER [12,13]. They are a very suitable low molecular weight cargo to be studied, as their autofluorescence resembles red fluorescent protein (RFP) and, as such, is amenable to microscopic tracking [14]. Direct import of anthocyanins into the vacuole is mediated by GST-ligandin transporters, namely TT19 of *Arabidopsis* [15]. Blocking the import of anthocyanins into the vacuole by inhibition of GST-ligandins (by buthionine sulfoximine, which inhibits glutathione synthesis or 1-chloro-2-4-dinitrobenzene, resulting in competitive inhibition of GST-ligandin transporters) did not result in the complete loss of intravacuolar anthocyanin accumulation, but in an increase of the number of AVIs [14]. Based on this observation, it was concluded that an alternative vesicle membrane container/vesicle (most probably a double-membrane)-mediated pathway exists and involves autophagic steps [14]. This hypothesis is supported by observations that several *atg* mutants (*atg5*, *atg9*, *atg10*) show anthocyanin accumulation defects, accompanied by a decreased number of neutral red stained bodies (NRSBs) and anthocyanin vesicular inclusions (AVIs) [7]. Despite these observations, the formation of intravacuolar bodies still remains enigmatic, as well as the possible cargoes transported.

## 3. Autophagic Tubes in Plants: Microautophagy with Macro Consequences?

In grapevine or maize, it was noticed many times that anthocyanins first start to accumulate in small tubular or vesicular bodies, later developing into larger bodies by fusion or vacuolar autophagy [16–19]. Similar tubular structures have been also observed in *Eustoma grandifolium* [20] or apple skin [21]. These bodies often contain numerous smaller compartments, indicating the complexity of the whole pathway. It has been speculated many times that the tubular structures accumulating anthocyanins form a network, strongly resembling the ER or ER-derived vesicles. Evidence for this was shown by co-localization of the GFP-HDEL (ER retention amino acid peptide fused to green fluorescent protein) with anthocyanin autofluorescence [14,17].

**Figure 2.**
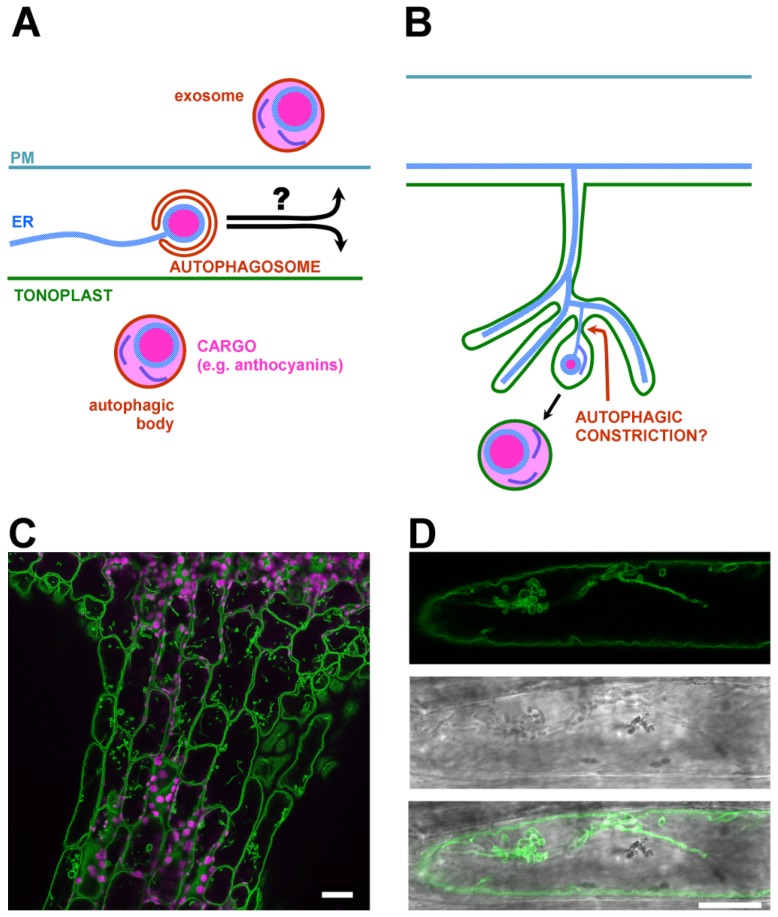
(**A**) A model of autophagic phytochemical transportation based on [7,14,17,29]; (**B**) An alternative proposed model with the involvement of microautophagy; (**C**) The tonoplast marker δTIP-GFP (tonoplast aquaporin δTIP fused to GFP) in the hypocotyl of a five-day-old *Arabidopsis* seedling with induced anthocyanin synthesis (+0.1 mM naringenin, 8 h) illustrates the abundance of tubular structures within tonoplast. Plastids are in magenta; and (**D**) Detailed view; the same as **C**. Scale bars represent 20 μm.

On the other hand, the position of these bodies and tubes in the tonoplast invaginations deep inside the vacuole and the dependence of AVI formation on autophagic machinery indicate that they may be a plant version of autophagic tubes, previously described in yeast [22]. Here, we would like to propose a hypothesis that phytochemical accumulation mechanisms in plants are partly mediated by autophagic tubes (Figure 2). Autophagic tubes represent a specific starvation-induced form of microautophagy. The tubes are branched, containing transmembrane protein-rich necks and lipid rich tips [22]. From these tips, intravacuolar autophagic bodies are budding. Surprisingly, not much is known about analogous tubes in plants despite the fact that such structures clearly exist (see Figure 2, [23,24]). These tubules are clearly distinct from the spherical vacuolar blobs, which contain a small vacuole or vacuolar domain instead of the cytoplasm [25]. The lack of information about such plant structures may be due to the lack of appropriate molecular markers. As was documented by *in vitro* yeast vacuole reconstitution assays, uptake of the cargo by autophagic tubes is not dependent on Vam3p and Vam7p, which are required for macroautophagy, nor on any other conventional component of the homotypic vacuole fusion [26]. The mere fact that the tubes persist on isolated vacuoles also documents their independence of the microtubule cytoskeleton [22,26]. This was also confirmed by the minimal effect of microtubular drugs [27].

On the other hand, yeast autophagic tube uptake is dependent on the guanosine triphosphate (GTP), membrane potential and autophagic machinery [26]. The precise role of autophagic machinery in autophagic tunnel formation is not known. It has been speculated before that it may be the membrane bending and shaping, associated with autophagy or the pinching off the budding autophagic body, which is also the only rapamycin-sensitive step [27]. It is tempting to speculate that the negative membrane curvature needed for such bending may be mediated by Exo70 proteins, as was recently shown in mammalian cells [28].

The model we are proposing here, in which ER subdomains (filled by phytochemicals) develop within autophagic tunnels (Figure 2B), explains many phenomena observed previously; for example, globular and fibrillar structures of AVIs, decreased AVI formation in autophagic mutants or the variety of shapes and sizes observed. Furthermore, a similar lipid composition of the AVI membrane and tonoplast [16] suggests that a form of microautophagy is responsible for the formation of these compartments. In this context, the effect of 3-methyl adenine, an inhibitor of Class III phosphatidylinositol 3-kinases on AVI formation [14], is also expected, as the source of the membrane for the autophagic tunnel is the tonoplast itself.

The physiological function of such tubular cellular compartmentalization might be that such tubules, being narrow and dead-ended, represent a location protected from the continuous cytoplasmic streaming, so that multiple enzymes attached to the cytosolic face of the ER might have an undisturbed, higher, local substrate/intermediate concentration. Moreover, with a turgid vacuole and relatively limited space in the cytoplasm, invasion of ER domains into the vacuole would also provide more surfaces for the massive synthesis of phytochemicals.

The presence of the ER in the autophagic tunnels is very likely, since the ER surface is the main site where anthocyanin synthesis occurs. Furthermore, anthocyanin transporter TT19 has been observed in the ER membrane [15]. As was convincingly shown, ER autophagy is induced by ER stress, with ER being present within the autophagic bodies, demonstrating that there is a mechanism for interaction between the macroautophagic structures and the ER [29]. However, it was not yet tested if the macroautophagy is the only mechanism responsible for ER degradation or if autophagic tubes contribute to the ER degradation.

## 4. Endoplasmic Reticulum Initiated Autophagy as a General Pathway for the Delivery of Secondary Metabolites and Phytohormones into the Vacuole and Apoplast?

Anthocyanins are just one example of many possible cargoes that could use the autophagic pathway. What other molecules can we expect to be transported in a similar fashion? There are many low molecular weight substances derived from or related to phenylpropanoid metabolism—structurally so similar to anthocyanins, that it is reasonable to assume that at least some of them might be also transported in similar autophagy-related membrane containers. It was observed already, more than ten years ago, that phytochemicals with distinct autofluorescence accumulate within membranous bodies, which are differentially targeted to the vacuole or apoplast [30]. Some of these bodies resemble the branched tubular structures mentioned above. We will start our discussion here with salicylic acid (SA). SA accumulates in several autophagy mutants, and SA and its agonist, benzo-(1,2,3)-thiadiazole-7-carbothioic acid, were shown to induce autophagy. This SA accumulation is obviously directly involved in the early senescence/hypersensitive response (HR) lesion phenotype deviation of autophagy mutants. It has been shown that SA accumulated in the autophagy mutants functions via an NPR1 (Nonexpressor of pathogenesis-related proteins 1) protein SA signaling hub [31].

As mentioned above, the content of stressed ER (visualized by the HDEL ER retention signal fused with GFP) is transported into the central vacuole in an autophagy-dependent (namely, ATG18) manner. This process is triggered by an ER stress sensor, IRE1b; however, it does not require bZIP60 [29]. Consistently, both IRE1b and IRE1a are upregulated by SA and mediate SA-induced unfolded protein response [31].

With respect to SA, it is important that it not only induces the autophagy machinery, but it was shown at the same time that autophagy itself acts in a regulatory negative feedback loop to suppress SA-induced responses, due to the suppression of SA signaling [31]. The mechanism of this suppression is, however, unknown. Most likely, this is due to detoxification of the cytoplasm from reactive oxygen species and, probably, SA itself. As SA is produced by chloroplasts, also chlorophagy may be the most important aspect of this negative feedback loop eliminating SA-loaded plastids [32]. This would also allow a satisfactory explanation of why most of the autophagic mutants show SA hyperaccumulation-dependent early senescence/ectopic hypersensitive response phenotypes even without stress [9,23,31]. We are currently testing in our laboratory a hypothesis that in *Arabidopsis*, autophagy is directly involved in the containment and catabolism of hyperaccumulated SA itself.

An interesting example of secondary metabolite compartmentalization, which resembles chlorophagy, represents tannosomes recently described in *Vitis* and *Gingko.* Here, tannins are synthetized in the chloroplast thylakoids, producing derived compartments: tannosomes. These are packaged together in the shuttle membrane body, which is then internalized in the central vacuole by a microautophagy-like mechanism. Therefore, there are three membranes separating tannins from the vacuolar lumen, preventing tannins from polymerizing in the vacuolar sap, causing protein denaturation [33].

ER stress, anthocyanin synthesis and general secondary metabolism is switched on by stress situations, and such stresses are quite common outside the growth chamber in nature. It is well known that suboptimal growth conditions or stress support the general accumulation of many secondary metabolites, with wound-induced jasmonic acid (JA) being a general stimulator of secondary substance metabolism [34–36]. Compartmentalization of secondary metabolite synthesis within plant cells is known only partly, and it is especially difficult to distinguish between cytoplasm *vs*. ER surface-localized biosynthetic machineries, as published in many biochemically-poised reports. However, there are clear hints that the anthocyanin autophagy-related vesicular import pathway from ER into the vacuole is not at all alone as an exception, and many other secondary metabolites might be transported via this pathway. It is well established that many secondary metabolites are produced via metabolons organized around specific ER domains with anchored cytochrome P450 monooxygenases [37–40]. This is true, e.g., for the phenylpropanoid/flavonoid and cyanogenic glucoside pathways [38]. Such ER-bound metabolons, proven for flavonoid and isoflavonoid synthesis, along with analyses of several autophagy-related *Arabidopsis* mutants (including *exo70B1*), indicate strongly that such a specific metabolon-rich domain of ER might possibly, at the same time, function as an initiation domain for autophagosome biogenesis. This is indicated also for alkaloids; for instance, for benzylisoquinoline alkaloids represented by sanguinarine. Sanguinarine is an antimicrobial alkaloid, which is synthesized in the ER and accumulated in poppy vacuoles in a GA-independent manner after elicitor treatment. After elicitation, large, dilated containers form from the lamellar ER and directly fuse with the vacuole [37]. Furthermore, a crucial part of the indole alkaloid (e.g., vinblastine) precursor pathway seems to be synthesized by the ER-anchored machinery in *Catharanthus roseus* [37]. As the autophagy-related process is inherently able to load into the nascent membrane containers, not only in the domain of ER, but also proximal pieces of cytoplasm, including solutes, it has the capacity to transport also into the vacuole those low molecular weight substances that are not directly synthesized in or on the ER surface. In this respect, it should be very informative to systematically study the secondary metabolite status of autophagy mutants.

In the case of anthocyanins (but, possibly, also cyanogenic glucosides and glucosinolates), it seems that important detoxifying glycosyl transferases are also a part (though only weakly associated) of ER bound metabolons [41,42]. It is known that SA is mostly modified in the cytoplasm into SA *O*-β-glucoside (SAG) by SA glucosyltransferases, and this form accumulates in the vacuole (for review including also other modifications, see [43]). The mechanism of a hypothetical putative SA/SAG loading into the autophagy-related membrane containers might include ER-localized transporters and ER-derived compartments. The possibility that SA itself is also downregulated by the pathway it induces would explain, in a straightforward way, the negative feed-back loop discovered by [31]. Similarly, abscisic acid glucosyl ester (ABA-GE) might be a cargo for such an autophagy-related transportation mechanism. Interestingly, the place of synthesis of ABA-GE remains unknown, but it is stored mainly in the vacuole and ER via several low affinity pathways [44]. However, AtBG1, a β-glucosidase that releases active ABA, is primarily localized to the ER and has a conserved ER retention signal [45]. This implies the possibility that not only ABA-GE, but also AtBG1 might be imported into the vacuole by the autophagy-like pathway, similar to GFP-HDEL [29]. In the *exo70B1* mutants, we have spotted also unusual accumulation of ABA, among some other regulatory substances [45].

It is important to note that loading into the autophagy-like membrane containers does not necessitate any cargo selection mechanism other than the formation of the ER domain endowed with specific synthetic, glycosylation or transporter activity, as is the case exemplified by prolamins and anthocyanins [46]. The organization of most of secondary metabolites producing metabolons around ER-anchored cytochromes P450 (CYPs; see above) make it possible to link/connect secondary metabolite production directly to autophagy-related membrane containment of potentially self-intoxifying substances in ER-derived autophagosomes, both macro- and micro- autophagically created by the “invagination” of tonoplast driven by specific ER domain (see above; [47]). This might include also some other phytohormones, such as auxin, which might be delivered from ER to the vacuole via these pathways after the ER accumulation driven by the “short” PIN proteins (PIN from PINOID Arabidopsis proteins—auxin efflux carriers) [48].

## 5. Putative Autophagy-Related Export of Secondary Metabolites to the Apoplast/Cell Wall

Autophagy-related transports in eukaryotic cells are known to be directed not only into the vacuole, but also to the outside of the cell [49,50]. In plants, this “outside” is very dynamic and, for the life of a plant cell, the crucial apoplastic space of cell walls [51]. First of all, it is well established that anthocyanins accumulate not only inside the vacuole, but also in the cell wall [52]; how they are delivered there is currently unknown, but AVI-like autophagosomes derived from the same ER-related autophagic process might, under specific circumstances, fuse also with the plasma membrane (PM), releasinganthocyanin-containing exosomes [53,54].

The antimalaric sesquiterpene, artemisinin, is synthesized in the cytoplasm from general isoprenoid precursors, and the important enzymes involved are localized at the ER [55]. Its accumulation in the apoplastic space of *Artemisia glandular* trichomes might well represent a putative example of autophagy-related export of secondary substances via exosomes.

The famous anti-cancer, anti-viral and healing red naphthoquinone shikonin accumulates in the roots of the *Boraginaceae* family. In model *Lithospermum erythrorhizon* cells and hairy root cultures after synthesis in the ER, the membrane containers with shikonin are bound to the PM [56,57], allowing one to speculate also about a possible autophagy-related mechanism of this process. The most vivid example of possible autophagy-related exosome secretion was provided in maize cells; upon the induction of the P1 transcription factor, autofluorescent membrane-bound bodies (possibly ER derived) accumulate in the cytoplasm and fuse with the PM, releasing their autofluorescent content into the cell wall in an exosome-like manner [30]. Phenylpropanoids, such as hydroxycinnamic acid derivatives, are found in the cell walls esterified to the wall polysaccharides; they are synthesized at the ER, and from there, they are released in a small membrane vesicles, which aggregate into bigger structures, fusing with the PM and releasing the content into the apoplast [58]. Furthermore, the biogenesis of these structures might be explained easily by autophagy-related process. It is well established that in the epidermal cells of leaves invaded by pathogens, the phenylpropanoid content in the wall increases; and they contribute also significantly to the general phytoalexin accumulation inside the defensive papillae. The mechanism of this accumulation is clearly indicated as multivesicular bodies (MVB) exosomes based [59], and obviously, for the biogenesis of such exosomes, the autophagy-related process might contribute significantly [9,53]. Based on these examples, it seems also reasonable to allow the possibility that similar pathways might operate also in cuticle biogenesis [60].

It might be concluded here that for both secondary metabolites accumulated in the vacuole, as well as in the cell wall/apoplast, autophagy-related membrane vesicle/container transport is a plausible alternative mechanism of delivery, apart from direct membrane transporter delivery.

## 6. Conclusions

We summarize in this review evidence that the direct transportation of ER-derived membrane containers to the vacuole or apoplast/cell wall, bypassing GA-TGN, has been reported many times in many different contexts, also in relation to different representatives of secondary metabolites in plant cells. Recently published studies suggest clearly an autophagic character of at a least subset of these processes. That the main source of the autophagic membrane is the ER itself is not yet generally accepted for plants, despite the fact that there is solid evidence of such a process in Opisthokonts and most of the published data point in this direction also in plants. Old and recent evidence for a decisive role of direct ER to vacuole transport in lytic vacuole biogenesis [10,61], along with the unexpected engagement of the exocyst subcomplex in autophagy-related direct ER to vacuole membrane transport [9], indicates a possibility that exocyst dynamics might be involved in the coordination between PM and tonoplast biogenesis (discussed in our recent review [53]). Here, we propose that direct ER-initiated autophagy is the simplest, most parsimonious way how to explain and interconnect the observations from cereals, vine and *Arabidopsis*, from anthocyanin accumulation studies and protein storage vacuoles and to interpret the multiple membrane topologies described, including extensive secondary metabolites containing tonoplast invaginations, which we interpret in the context of similar microautophagy-related tubular structures in yeast vacuoles. Rapid detoxification of the cytoplasm from various secondary metabolites is essential for plant survival, even under normal conditions. We therefore speculate that many secondary metabolites, including phytohormones, such as auxin, ABA and salicylic acid, but also alkaloids, might be the cargo of this pathway, as anthocyanins are. Autophagy-related membrane trafficking might significantly contribute not only to secondary metabolite turnover and accumulation, but also phytohormonal regulation in developmental and environmental contexts.
